# The Gut Microbiome in Anxiety Disorders

**DOI:** 10.1007/s11920-025-01604-w

**Published:** 2025-04-12

**Authors:** Mary I. Butler, Sarah Kittel-Schneider, Jolana Wagner-Skacel, Sabrina Mörkl, Gerard Clarke

**Affiliations:** 1 Department of Psychiatry and Neurobehavioural Science, University College Cork, Cork, Ireland; 2 APC Microbiome Ireland, University College Cork, Cork, Ireland; 3 Department of Psychiatry, Psychotherapy and Psychosomatic Medicine, University Hospital of Würzburg, Würzburg, Germany; 4 Division of Medical Psychology, Psychosomatics and Psychotherapeutic Medicine, Medical University of Graz, Graz, Austria

**Keywords:** Gut microbiome, Gut microbiota, Gut-brain axis, Generalised anxiety disorder, Social anxiety disorder, Panic disorder, Agoraphobia

## Abstract

**Purpose of review:**

We aim to update readers on the latest evidence regarding the role of the gut microbiome in generalized anxiety disorder (GAD), panic disorder (PD), agoraphobia, and social anxiety disorder (SAD). This review summarises the literature on microbiome composition and function in these conditions, provides insights about causality and mechanisms and evaluates current evidence for microbiome-based interventions in anxiety disorders.

**Recent findings:**

Most studies exploring the microbiome in anxiety disorders are small, cross-sectional studies. Nevertheless, some consistent findings emerge. Bacterial taxa such as *Eubacterium, Coprococcus* and *Faecalibacterium* may be depleted in GAD. Studies in PD and SAD are scarce and, to our knowledge, there have been no studies conducted in agoraphobia. Probiotics may help reduce anxiety symptoms, although the majority of studies have been in non-clinical cohorts.

**Summary:**

Large, prospective studies are required to further elucidate the role of the microbiome-gut-brain axis in anxiety disorders. Microbiome-based interventions hold promise, but randomised controlled trials in clinical populations with relevant diagnoses are now warranted and urgently required.

## Introduction

Anxiety disorders are a heterogeneous group of conditions with high levels of comorbidity with other mental disorders [1]. The most common anxiety disorders in adults seen in clinical practice include generalised anxiety disorder (GAD), social anxiety disorder (SAD), panic disorder (PD) and agoraphobia. Post-traumatic stress disorder (PTSD) was previously categorised as an anxiety disorder but is now separately classified as a trauma- and stressor-related disorder [2]. Similarly, obsessive–compulsive disorder, once classified as an anxiety disorder, is now conceptualised as a unique and distinct condition [2]. In this review, we focus on the aforementioned anxiety disorders: GAD, SAD, PD and agoraphobia. These conditions are highly prevalent, chronic and often significantly disabling [1]. Anxiety is also frequently comorbidly expressed in other disorders such as major depression [3] and irritable bowel syndrome (IBS) [4]. Despite this, anxiety disorders are frequently under-diagnosed and under-treated [5]. While first-line treatment options such as serotonergic medications or cognitive-behavioural therapy can be effective, treatment resistance is high and clinical needs remain unmet for a significant proportion of sufferers [5]. Developing new precision treatment approaches is vital and the microbiota-gut-brain axis represents an attractive new therapeutic target.

The gut-brain axis – the bidirectional communication system between the gut and brain – comprises neural, immune, metabolic and endocrine signalling pathways. The gut microbiota – the trillions of bacteria inhabiting the gastrointestinal system – are now recognised as being key players in gut-brain axis communication [6]. An appreciation of the importance of the microbiome-gut-brain axis in the regulation of the stress-response and anxiety behaviours began with animal studies using various techniques, including germ-free and antibiotic-depleted animals, pathogenic bacterial infections, probiotic and prebiotic interventions and faecal microbiota transplantation [7]. This preclinical work demonstrated the impact of the gut microbiome on many physiological pathways involved in the pathogenesis of anxiety disorders, including hypothalamic–pituitary–adrenal (HPA) axis responsivity, immune modulation, tryptophan-kynurenine metabolism, vagal nerve communication, brain-derived neurotrophic factor (BDNF) expression, neurogenesis and myelination, microglial function and neurotransmitter production [7–9].

We carried out a narrative review synthesizing the recent literature from human studies, along with major advances from preclinical studies. To identify relevant articles, we conducted keyword searches using Pubmed, PsychInfo and Scopus. We searched and selected peer-reviewed research articles that were written in the English-language and published between 1st Jan 2018 and 30th June 2024. This review focuses specifically on providing an up-to-date synthesis of the evidence for the role of the microbiome in patients with clinical anxiety disorders: GAD, PD, agoraphobia and SAD. We discuss the literature on the composition and function of the gut microbiome in clinical anxiety disorder cohorts, supported with some key observations from studies focused on anxiety symptoms. We also evaluate the current evidence for microbiome-based interventions in anxiety disorders.

## Anxiety Disorders: Neurobiology and Current Therapeutic Targets

Anxiety and fear are vital, evolutionarily conserved emotions which arise in response to potential or real threats. The neurobiological process by which such responses become excessive, prolonged and pathological, as is the case with anxiety disorders, remains poorly understood. A portion of the variance in susceptibility risk for anxiety disorders can be explained by genetic risk and the epigenetic impact of environmental factors such as trauma and chronic stress [10]. Basic neuroscience research suggests that impaired threat responses involve dysfunction of brain circuity that deals with attention, emotion, learning and memory, findings which have been effectively translated in humans using functional neuroimaging [5]. At a systems level, the hypothalamic–pituitary–adrenal (HPA) axis and peripheral immune system have been studied. Findings with regards to HPA activity at baseline and after stress stimuli are inconsistent in anxiety disorders [11, 12]. The reasons for such variability are unknown but may represent differences in the course and chronicity of the disorder, symptom severity and sex differences. Peripheral blood inflammatory markers appear to be altered in anxiety disorders but findings are preliminary and require further investigation [13, 14]. Neurotransmitter abnormalities have been implicated in anxiety, leading to the existing pharmacotherapy options which include serotonergic, noradrenergic and GABAergic medications [15]. More recent novel therapeutic drug targets involve the glutamatergic and endocannabinoid systems as well as various neuropeptides such as vasopressin, oxytocin, orexin [16]. Additionally, natural plant compounds and plant extracts are being studied for their anxiolytic potential. Those ‘phytochemicals’ which have demonstrated anxiolytic activity include *Piper methysticum* (kava), *Centella asiatica* (pennywort), *Humulus lupulus* (hops), *Ginkgo biloba* (maiden hair), *Matricaria chamomilla* (chamomile), *Melissa officinalis* (lemon balm), *Passiflora incarnata* (maypop), *Scuterllaria leriflora* (skullcap), *Valeriana officinalis* (valerian), *Withania somnifera* (ashwagandha), *Magnolia officinalis* (magnolia bark) and *Lavendula angustifolia* (lavender), among others [16]. Although the mechanisms underlying their effects are largely unknown, modulation of the GABA system is implicated [17]. Recent clinical guidelines for the treatment of mental disorders with nutraceuticals and phytoceuticals support the use of ashwagandha, galphimia and lavender in the treatment of anxiety disorders [18].

## Microbiome-Gut-Brain Axis in Anxiety Disorders: Focus on Signalling Pathways

**Fig. 1:**
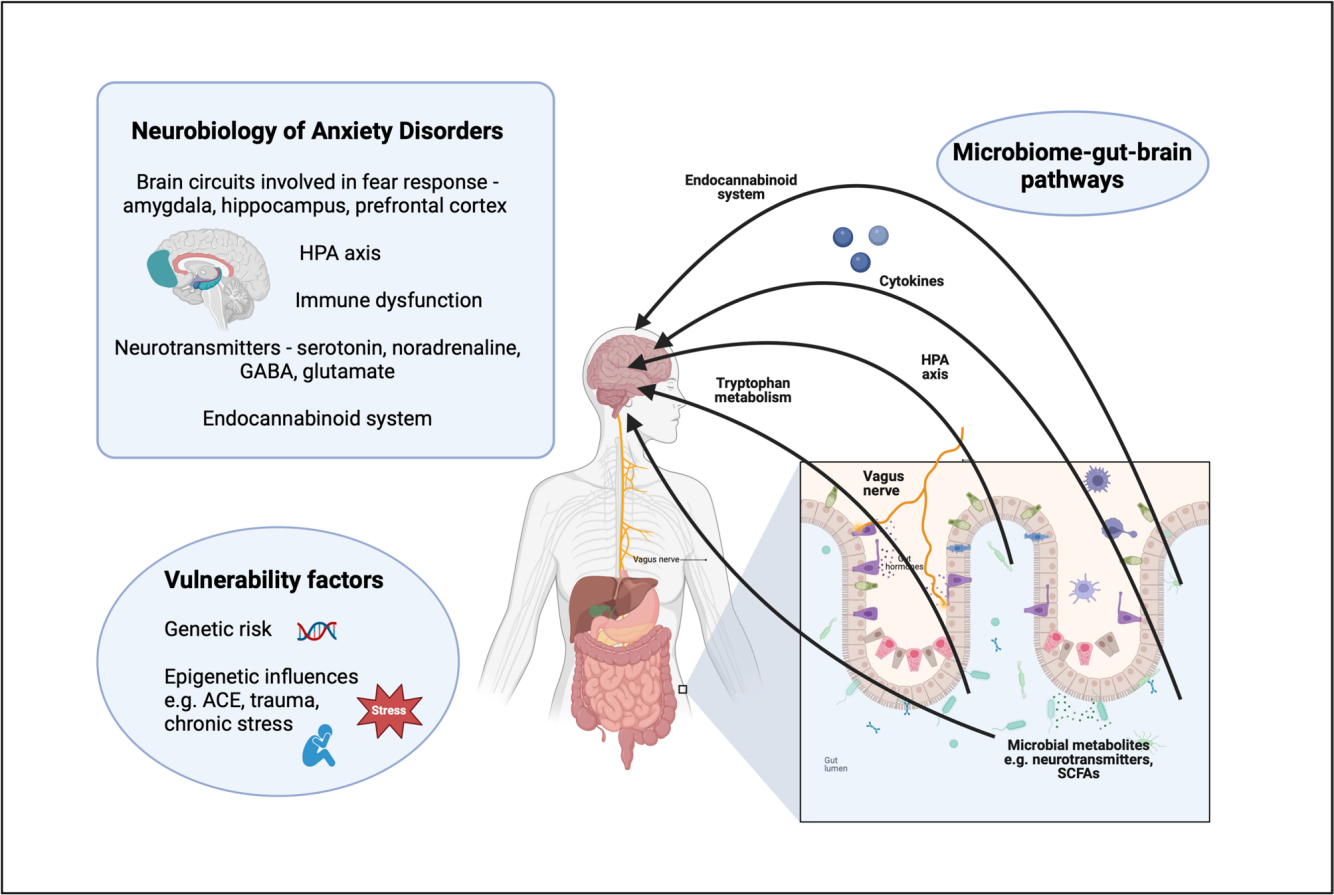
summarises the known aetiological risk factors and underlying neurobiological abnormalities in anxiety disorders. It demonstrates the various microbiome-gut-brain signalling pathways which encompass those physiological systems involved in the pathogenesis of anxiety and stress-related conditions. *(ACE: adverse childhood events, GABA: gamma-aminobutyric acid HPA: hypothalamic–pituitary–adrenal axis, SCFA: short-chain fatty acids)*

Microbiome-gut-brain signalling pathways encompass a wide variety of the physiological systems implicated in anxiety disorders (Fig. 1). A large body of evidence exists supporting a role for the microbiome in the early development and ongoing regulation of stress responsivity. Preclinical studies indicate that disruption in microbiome composition early in life using antibiotics, bacterial infections, Caesarean-section births, various acute and chronic stress exposures and other environmental influences can result in significant, enduring alterations in HPA axis activity and stress response [19]. Similar preclinical methods have been used to demonstrate the substantial immunomodulatory properties of the gut microbiome at birth and throughout the lifespan [20]. Although the stress response system and immune function are perhaps the most studied gut-brain signalling pathways, numerous other gut-brain cross-talk mechanisms are at play. Microbial regulation of the metabolism of tryptophan is important in stress-related psychiatric disorders, both due to the role of tryptophan as a precursor to serotonin and its metabolism via the neuroactive kynurenine pathway [21]. We have previously demonstrated an alteration in kynurenine pathway metabolites in social anxiety disorder [22]. Microbial metabolites are important mediators of gut-brain communication. Short-chain-fatty-acids (SCFA) are key metabolites produced by bacterial fermentation of fibre and exert both direct and indirect effects in the brain. They influence intestinal barrier integrity [23], which we have recently found to be disrupted in people with social anxiety disorder (unpublished data). SCFAs augment blood brain barrier tight junction expression and protect against central neurotoxin infiltration in addition to regulating host GI immunity and peripheral immunity, modulating HPA axis response and directly impacting the concentrations of neurotransmitters and neurotrophic factors in the gut lumen [23]. Moreover, exciting work has demonstrated that the SCFA, acetate, is one of the mechanisms underlying the known impact of the gut microbiota on the maturation and function of brain microglia [24, 25]. The endocannabinoid system, which is thought to buffer against many of the effects of stress [26], is also influenced by gut microbiota composition [27]. It has been demonstrated that the impact of the gut microbiota on depressive-like behaviours in mice is mediated by the endocannabinoid system [28]. An additional mechanism of gut-brain signalling is via hippocampal neurogenesis. Faecal microbiota transplant and probiotic supplementation in animal models has demonstrated that microbial signalling can alter levels of hippocampal BDNF and influence adult hippocampal neurogenesis, with subsequent consequences for cognition, stress and emotional regulation [29]. The myriad pathways through which the gut microbiota impact brain function and behaviour highlight the complexity of gut-brain interplay and the many potential avenues for the development of anxiety disorders.

## Microbiome Composition and Function in Clinical Anxiety Disorders

Several studies have investigated gut microbiome composition and/or function in GAD, PD and SAD. A summary of these studies and their findings are outlined in Table 1. While most studies are heterogeneous and of small sample size requiring cautious interpretation, some consistent and interesting trends in associations with specific bacterial taxa can be seen, including some that may be transdiagnostic across a variety of psychiatric conditions [30]. To our knowledge, no studies have been undertaken in people with agoraphobia to date.

**Table 1:** Summary of cross-sectional and prospective studies investigating microbiome composition and function in generalised anxiety disorder, panic disorder and social anxiety disorder

Author (Country of study)	Study Details	Microbiome source	α diversity	β diversity	Compositional differences	Functional differences	Other
(Jiang et al*.*, 2018) (China)	Cross-sectional study of 40 patients with active GAD and 36 controls	Faecal microbiome	Reduced richness (number of OTUs) in GAD but no difference in Chao, Shannon or Simpson indices	Difference seen between GAD and HC groups	Phylum: *Firmicutes spp* decreased in GAD *Fusobacteria* and *Bacteroidetes spp* over-represented in GAD *Faecalibacterium, Eubacterium rectale,* *Sutterella, Lachnospira,* and *Butyricicoccus* enriched in the controls *Ruminococcus gnavus* and *Fusobacterium* increased in GAD	Not explored	
	Cross-sectional analysis of subgroup of 12 treatment-naïve GAD patients and 17 controls	Faecal microbiome	Reduced alpha diversity in GAD (reduced number of OTUs, ACE, Chao and Simpson indices)	Trend towards a difference between the two groups (p = 0.06)	*Faecalibacterium, Eubacterium rectale, Roseburia,* *Subdoligranulum*, and *Lachnospira* increased in controls *Bacteroides, Escherichia-Shigella, Ruminococcus gnavus, Lactobacillus* and *Fusobacterium* increased in GAD	Not explored	
	Prospective study of subgroup of 9 patients in both the active and remissive states of GAD	Faecal microbiome	No difference between groups	No difference between the two groups	*Bacteroides spp*., showed a decrease in the remissive state *Faecalibacterium, Eubacterium rectale* and *Sutterella* were more frequent in the remissive state compared with the active state	Not explored	
(Chen et al*.*, 2019) (China)	Cross-sectional study of 36 patients with active GAD and 24 controls	Faecal microbiome	Reduced number of OTUs and ACE in GAD patients. No difference in Chao, Shannon or Simpson indices	Difference seen between GAD and HC groups	Phylum level: Firmicutes enriched in controls Class: Tenericutes, Mollicutes_RF39_norank, Mollicutes enriched in HC Order: Betaproteobacteriales, Enterobacteriales enriched in GAD Family: Burkholderiaceae, Enterobacteriaceae, Bacteroidaceae enriched in GAD Prevotellaceae, Muribaculaceae, Ruminococcaceae_UCG-014, Lachnospiraceae NK4A136 group, Ruminococcaceae NK4A214 group enriched in HC Genus: *Tyzzerella, Hungatella*, *Escherichia–Shigella*, and *Bacteroides* enriched in GAD *Prevotella* 9, *Dialister, Eubacterium_coprostanoligenes* group, *Subdoligranulum, Megamonas, Agathobacter, Coprococcus 1,* *Clostridium innocuum group, Buchnera,* *Eubacterium xylanophilum group, Coprococcus 3, Eubacterium ruminantium group, and Acinetobacter* more abundant in HCs	Not explored	Abundances of E*ubacterium_coprostanoligenes*_group, Ruminococcaceae_UCG-014, and *Prevotella*_9 correlated negatively with the anxiety severity and positively with anxiety reduction Abundances of *Bacteroides* and *Escherichia-Shigella* were positively associated with anxiety severity
(Mason et al*.*, 2020) (USA)	Cross-sectional study with four groups: • 10 healthy controls • 38 MDD and anxiety (comorbidity group) • 8 anxiety only • 14 MDD only (Anxiety = either GAD or anxiety-NOS diagnoses) Analysis looked specifically at certain bacterial genera: *Eubacteria* (total bacteria, EUB), E*nterobacteriaceae* (ENTERO), *Eubacterium rectale/Closteridium group* (EREC, Clostridial cluster XIVa), *Lactobacillus/Enterococcus Group* (LACT)*, Bacteroides* (BACT), and *Clostridium leptum group* (CLEPT, Clostridial cluster IV)	Faecal microbiome	No difference between the four groups	No difference between the four groups	EUB reduced in comorbidity compared to HC BACT reduced in comorbidity compared to MDD CLEPT reduced in MDD compared to HC and comorbidity abundance of EREC and CLEPT were negatively associated with the severity of depression and the severity of anxiety. EUB was also associated with the severity of anxiety	Not explored	Authors concluded that reduced or absent *Clostridia* was consistently seen in those with depression, independent of the presence of anxiety. Conversely, reduced *Bacteroides* may be more associated with the presence of anxiety, independent of the presence of depression
(Dong et al*.*, 2021) (China)	Cross-sectional study with three groups: • 23 patients with MDD • 21 with GAD • 10 healthy controls	Faecal microbiome	GAD group showed a significant reduction in microbiota richness (ACE and Chao) and diversity (Shannon) as compared with controls. No significant difference in richness and diversity between controls and MDD		Genus level: Abundances of *Sutterella* and *Fusicatenibacter* were significantly lower in MDD relative to controls Abundances of *Fusicatenibacter* and *Christensenellaceae*_R7_group were significantly lower in GAD than in controls Abundance of *Sutterella* was significantly higher whereas that of *Faecalibacterium* was significantly lower in GAD relative to MDD	69 different Kegg Orthologues between the three groups	Christensenellaceae_R7_group negatively correlated with total score of HAMD In GAD group *Faecalibacterium* negatively correlated with total plasma cortisol
(Brushett et al*.*, 2023) (Netherlands)	Cross-sectional study using data from 7,656 participants of the Dutch Lifelines population cohort • MDD (*n* = 70) • Dysthymia (*n* = 156) • Any anxiety disorder (*n* = 385) • GAD (*n* = 339) • Social phobia (*n* = 70) • PD (*n* = 11) • Controls (*n* = 5,522) Any anxiety disorder group included GAD (*n* = 339), social phobia (*n* = 70), PD (*n* = 11) All results adjusted for psychotropic drug (PTDs) use	Faecal microbiome	No significant associations observed between any internalising disorders or PTDs and alpha diversity	All internalizing disorders moderately but significantly explained the gut microbiome variation between participants, whether adjusted or unadjusted for SSRI or PTD use	GAD associated with decreased *Coprococcus eutactus* Any anxiety disorder (GAD, SAD, PD) associated with decreased *Coprococcus eutactus and B Bifidum* and increased *Clostridium lavalense*	No significant association between any anxiety disorders or gut-brain modules	All results adjusted for psychotropic drug (PTD) use
(Xie et al*.*, 2021) (China)	Cross-sectional study with patients with PD (*n* = 26) and healthy controls (*n* = 40)	Oral Microbiome	PD exhibited higher alpha diversity (based on observed species, Chao, Simpson and Shannon indices)	Difference seen between PD and control groups	Relative abundance of 61 genera differed between groups. (Top 20 differential genera shown here) *Akkermansia, Atopobium, Bacteroides, Barnesiella, Capnocytophaga, Corynebacrtium, Lactobacillus, Parvimonas, Prevotella, Saccharibacteria_genera_incertae_sedis, Selenomonas, SR1_genera_incertae_sedis, Schwartzia, Veillonella* enriched in the PD group *Rothia, Alloprevotella, Fretibacterium, Alistipes, Campylobacter, Escherichia/Shigella* increased in controls	29 different KEGG orthologues between the 2 groups	
(Lin et al*.*, 2023) (China)	Cross-sectional study of 40 patients with perimenopausal PD and 40 healthy controls	Faecal microbiome	Reduced α-diversity (richness) in the gut microbiota of perimenopausal PD patients. (OTU index and Shannon index lower in PPD but Simpson index higher)	Difference seen between PD and control groups	Phylum: Bacteroidetes and Verrucomicrobia more abundant in PDD. Firmicutes and Actinobacteria were less abundant in perimenopausal PD Genus level: *Bacteroides, Phascolarctobacterium, Parabacteroides, Alistipes, Paraprevotella, Sutterella, Akkermansia, Megasphaera, Veillonella, Bilophila, Flavonifractor, Oscillospira, Oscillibacter, Odoribacter, Butyricimonas,* and *Desulfovibrio* increased in PDD *Faecalibacterium, Blautia, Pseudobutyrivibrio, Subdoligranulum, Roseburia, Coprococcus, Bifidobacterium, Clostridium_sensu_stricto_1, Streptococcus, Dorea, Anaerostipes, Anaerotruncus, Collinsella,* and *Turicibacter* enriched in controls	Not explored	
(Butler et al*.*, 2023) (Ireland)	Cross-sectional study of 36 adults with SAD and 18 controls	Faecal microbiome	No difference between groups	Difference seen between SAD and control groups	*Anaeromassillibacillus* and *Gordonibacter* elevated in SAD patients *Parasuterella* more abundant in controls	Increased abundance of GMM ‘aspartate degradation I’ pathway in SAD No differences in GBMs	

*ACE* Abundance-based Coverage Estimator, *GAD* Generalised Anxiety Disorder, *GBM* Gut-Brain Modules, *GMM* Gut Metabolic Modules, *MDD* Major Depressive Disorder, *OUT* Operational Taxonomic Unit, *PD* Panic Disorder, *PTD* Psychotropic Drug Use, *SAD* Social Anxiety Disorder

The question is raised as to which level of taxonomic classification is most useful when exploring disease-associated microbiome differences [31–33]. Some studies report differences across all taxonomic levels from phylum down to species-level. However, others only report differences in genus and species level. A recent study that explored the classification of six diseases using a machine learning algorithm and gut microbiome data reported that the performance of classification is improved by using a lower taxonomy level; the highest performance was observed at the genus level [34]. This may be because lower taxonomic ranks show greater correlation with the faecal metabolome than higher order taxonomic groups and thus provide greater insights regarding crosstalk between the intestinal microbiome and the host [35]. We will, therefore, focus on genus- and species-level findings here.

### Generalised Anxiety Disorder

Gut microbiome richness (observed number of species, Operational Taxonomic Units (OTUs) or Amplicon Sequence Variants (ASVs)) is reduced in GAD [36–38]. However, alpha diversity measures that also consider taxa evenness, e.g., Shannon and Simpson indices, do not appear to be different to healthy controls [36, 37].

*Coprococcus* and *Faecalibacterium,* other prominent butyrate-producers in the human gut, also appear important in anxiety disorders. Chen et al. [37] reported depleted levels of *Coprococcus* in GAD patients, with a similar finding seen in perimenopausal PD [42]. Such outcomes are consistent with a large cross-sectional study which used data from 7,656 participants of the Dutch Lifelines Microbiome Project (DMP) cohort [43]. This study collected metagenomic sequencing data along with a wide range of biomedical, socio-demographic, behavioural, physical and psychological metadata. They assessed for the presence of a range of psychiatric disorders based on Diagnostic and Statistical Manual of Mental Disorders (DSM-IV-TR) criteria using a standardized diagnostic interview. Their analyses pertaining to anxiety disorders involved those with ‘any anxiety disorder’ (which they defined as GAD, SAD or PD) or those with GAD only. They did not analyse SAD or PD separately given the small numbers. The study aimed to explore the associations of the gut microbiome with anxiety and depressive disorders while adjusting for the use of psychotropic medications. This was an important study, given the potentially confounding impact of psychotropic drug use in many smaller cross-sectional studies. The study reported that any anxiety disorder (defined as GAD, SAD or PD), and GAD analysed individually, were significantly associated with a decreased relative abundance of *Coprococcus eutactus,* even after adjusting for psychotropic drug use*.* The overall conclusion from this study was that mood and anxiety disorders rather than psychotropic drugs are associated with compositional gut microbiome differences relative to controls. *Faecalibacterium* is another bacterial group that repeatedly emerges in the literature about psychiatric disorders and the gut microbiome. One GAD study found it to be significantly depleted in the patient group [36] and another study in GAD patients reported that *Faecalibacterium* negatively correlated with total plasma cortisol [38]. Additionally, *Faecalibacterium* was more abundant is people with GAD in remission compared with the active state [36]. An important metagenomics study using a large microbiome population cohort (Flemish Gut Flora Project, n = 1,054) with validation in independent datasets (*n* = 1,070) has previously found *Faecalibacterium* and *Coprococcus* bacteria to be consistently associated with higher quality of life indicators [44].

It is interesting that certain compositional findings have been replicated in a number of GAD studies, however it is unlikely that such changes are highly specific to GAD. A recent meta-analysis of gut microbiome alterations across a wide variety of mental disorders found a transdiagnostic pattern of microbiota signatures as opposed to any evidence of disorder specificity [30]. Depleted levels of *Faecalibacterium* and *Coprococcus* and enriched levels of *Eggerthella* were consistently shared between major depressive disorder, bipolar affective disorder, psychosis (undefined) and schizophrenia, and anxiety.

While *Eubacterium, Faecalibacterium* and *Copcococcus* are depleted in GAD, several bacterial groups may be more abundant in these individuals. Genera associated with GAD included *Ruminococcus gnavus* and *Fusobacterium* [36]. In a subgroup analysis of treatment-naïve patients, *Escherichia–Shigella* and *Bacteroides* were also enriched [36]. These taxa were also elevated in a subsequent cross-sectional study where their abundance was positively correlated with GAD symptom severity [37]. Additionally, a high abundance of *Bacteroides eggerthii* immediately after a two-month frontline work period during the Covid-19 pandemic was associated with future PTSD symptoms [41]. *Bacteroides* are a complex group of bacteria. While they represent a significant proportion of human gut commensals, these gram-negative obligate anaerobes can be highly pathogenic [45]. Similarly, *Escherichia–Shigella* is another pathogen which is associated with several human diseases [46]. Stress-induced proliferation of *Escherichia coli* in mice is associated with increased anxiety-like behaviours, decreased hippocampal BDNF expression and elevated gastrointestinal and hippocampal inflammation [47]. No such bacterial taxa were enriched in GAD by the larger Bruschett et al. (2023) study, which used the Dutch Lifelines cohort data. This may be because psychotropic drugs were accounted for as well as differences in study design (such as small sample sizes, differences in microbiome preparation and analysis, etc.).

Data in relation to functional microbiome differences in GAD is limited. No association was found between any functional gut-brain modules (GBMs) and anxiety disorders in the Dutch Lifelines cohort study [43]. GBMs represent a database of manually-curated microbial pathways known to impact brain function, based on extensive literature review. Each GBM corresponds to a single neuroactive compound production or degradation process [44]. A small study exploring microbiome differences between GAD, MDD and a control group reported differences in 69 Kegg Orthologues between the three groups, thus suggesting some differences in predicted microbiome function [38]. However, the numbers in each group were small and this observation requires further replication.

### Panic Disorder and Agoraphobia

Studies exploring microbiome composition are limited in PD and, to our knowledge, have not been conducted in agoraphobia. A small cross-sectional Chinese study has investigated the oral microbiome in PD [48]. The oral microbiome was significantly more diverse in PD patients, and many taxa differences were observed between the patients and controls. The relative abundances of *Prevotella* and *Veillonella* were higher in the PD group. Authors reported a predominance of these taxa in periodontal disease, which is more likely in PD. Another small study has explored the gut microbiota in perimenopausal PD [42]. They reported reduced alpha diversity in perimenopausal PD patients. Similar to findings in GAD, butyrate-producing groups, including *Faecalibacterium, Copcococcus* and *Roseburia* were depleted in relative abundance in perimenopausal PD, while the genus *Bacteroides* was elevated.

### Social Anxiety Disorder

Our research group has recently reported the first findings on the gut microbiota in social anxiety disorder [49]. While there had long been interest in the gut microbiota in anxiety and stress regulation, a growing appreciation for the role of the microbiome in social development and behaviour has developed in recent years [50]. We investigated the composition and function of the gut microbiome in 32 patients with social anxiety disorder in comparison to a healthy control group. No differences were seen in alpha diversity. However, we found that overall microbiota composition, as measured by beta-diversity, differed between the SAD and control groups. Several taxonomic differences were seen at a genus- and species-level: the relative abundance of the genera *Anaeromassillibacillus* and *Gordonibacter* were elevated in SAD, while *Parasuterella* was enriched in healthy controls. *Anaeromassilibacillus* is a member of the Clostridiales order of bacteria, a group which appears to show altered abundance in many psychiatric disorders and may represent disease-shared microbial responses [51]. In relation to functional differences, the gut metabolic module ‘aspartate degradation I’ was elevated in SAD patients. This functional pathway is associated with tryptophan-kynurenine metabolism, which we have previously demonstrated to be altered in SAD [22].

In order to test the hypothesis that the microbiota plays a causal role in SAD, we subsequently used faecal microbiota transplantation (FMT), a method used to assess potential causality and mechanisms [52, 53]. This involved the transfer of the microbiota from patients with SAD to antibiotic-depleted mice recipients and assessment of the behavioural and biological impact of such microbiota alteration [54]. Interestingly, the mice who received the SAD microbiome demonstrated a specific heightened social fear response, a validated mouse model of SAD [55]. They performed normally across other tests evaluating general anxiety-like and depression-like behaviours, an important feature of the study highlighting specificity for social fear responses. Additionally, changes in central and peripheral immune function and oxytocin expression in the bed nucleus of the stria terminalis were evident in the SAD-FMT-recipient mice.

## Microbiome-Targeted Therapeutics in Anxiety Disorders

### Probiotics, Synbiotics and Prebiotics

Numerous systematic reviews and meta-analyses have explored the impact of probiotics and prebiotics on stress and anxiety symptoms over the past five years [56–61]. For the most part, probiotics appear to be a promising intervention for reducing stress and anxiety symptoms, although results can vary depending on study inclusion criteria and the type of probiotic used, since strain-specific effects and different mechanisms of action are likely. The vast majority of studies included in these meta-analyses were not patients with a formal clinical diagnosis of an anxiety disorder. Rather, they include various combinations of healthy volunteers, patients with IBS (a disorder of gut-brain interactions with significant psychiatric comorbidity including anxiety) or other medical conditions, subjects under stress or people with depression. A meta-analysis of 1146 healthy subjects found that probiotics reduced subjective stress and improved stress‐related subthreshold anxiety/depression levels, although no impact on cortisol levels was observed [58]. A larger meta-analysis of 29 randomised controlled trials (RCTs) (n = 2035 participants) found that probiotics and synbiotics were effective in reducing anxiety symptoms, but prebiotics had no effect [59]. This was similar to an earlier meta-analysis, which included 34 RCTs involving healthy subjects, medical patients (with a range of medical problems including IBS, multiple sclerosis, obesity, fibromyalgia, rheumatoid arthritis and laryngeal cancer) and subjects with MDD, which reported a small anxiolytic effect of probiotics but not prebiotics [56]. A recent meta-analysis included only patients who had a clinical diagnosis (MDD: n = 4 studies, GAD: n = 1 study) or healthy subjects who were under stress (academic stress: n = 4 studies, socially-evaluated cold pressor test (SCEPT) condition: n = 1 study) [57]. They reported that a probiotic reduced depression scores but not anxiety scores. A meta-analysis involving pregnant (n = 946) or lactating (n = 524) women reported that probiotics were effective in reducing both anxiety and depressive symptoms. This may be a particularly important group when it comes to increasing therapeutic options for anxiety and depression, given uncertainty about the potential impact of antidepressant exposure in pregnancy [62].

Only one randomised controlled trial to date has investigated the impact of a probiotic in GAD [63]. This Iranian study randomised 48 medication-free patients with GAD to receive either a placebo or multispecies probiotic (18*10^9^ CFU *Bifidobacterium longom, Bifidobacterium bifidum, Bifidobacterium lactis, Lactobacillus acidophilus*) in addition to 25 mg of Sertraline for eight weeks. The group receiving the adjunctive probiotic had significantly greater reductions in the clinician-rated Hamilton-Anxiety Rating Scale, although no differences were seen in Beck Anxiety Inventory or the State Trait Anxiety Inventory.

To our knowledge, there have been no clinical trials using microbiota-based therapies in SAD, PD or agoraphobia. However, a cross-sectional study of over 1000 university students found that higher intake of fermented foods appeared to be protective against developing SAD in those at higher genetic risk, as measured by trait neuroticism [64]. High intake of fermented foods may also be protective against general anxiety symptoms [65]. Fermented foods are an important source of potentially beneficial bacteria, generally containing various strains of lactic acid bacteria [66]. When consumed in high amounts by humans, certain fermented foods have anti-inflammatory effects [67] which may, in part account for the benefit in mental health.

### Dietary Interventions

Diet is a major determinant of microbiome composition [68] and a promising intervention for psychiatric disorders, recently reframed under the banner of Nutritional Psychiatry [69]. It is well recognised from population studies that a Mediterranean diet is protective against depression [70]. More recently, there is evidence that high adherence to a Mediterranean-type diet may also be protective against anxiety symptoms in both adults [71, 72] and older people [73], as well as being associated with lower odds and severity of anxiety disorders [74]. Conversely, a diet characterised by high-fat, high-sugar and low fruit and vegetable intake, characteristic of the ‘Western style’ pattern of eating, is associated with elevated anxiety symptoms [75].

The mental health benefits of a Mediterranean diet extend beyond prevention. In 2017, the SMILES trial demonstrated for the first time that a Mediterranean diet intervention could improve depressive symptoms in Australian patients with MDD alongside standard treatment including psychotherapy and/or pharmacotherapy. [76]. Several subsequent clinical trials, also in Australia, demonstrated similar findings in depressed patients [77–79]. To date, no clinical trials have specifically explored the Mediterranean diet as a therapeutic intervention in anxiety disorders. However, in the aforementioned SMILES trial, a reduction in the Hospital Anxiety and Depression Scale (HADS)-anxiety subscale score was reported as a secondary outcome.

Interestingly, a Mediterranean diet intervention results in an increased abundance of *Faecalibacterium* and *Roseburia* [80, 81] taxa, which are depleted in GAD and PD [36, 42]. A study from our research group recently investigated the impact of a ‘psychobiotic diet’ in healthy volunteers [82]. The ‘psychobiotic diet’ included aspects of the Mediterranean diet like fruits, vegetables, whole grains, legumes, and seeds, as well as fermented foods. After four weeks of adhering to this diet, subjects reported reductions in perceived stress which were greatest in those with high adherence. Dietary intervention remains a promising therapeutic strategy for anxiety disorders and needs to be explored further.

## Conclusions/Future Perspectives

It is an exciting time in neuroscience and psychiatry. The exponential increase of microbiome-gut-brain axis research over the past two decades has led to hope of new approaches for the treatment of anxiety. Given the growing burden of anxiety and stress-related disorders, along with the significant number of patients who do not respond fully to conventional treatments, alternative options and the availability of adjunctive approaches are vital. However, much work remains to be done. A key priority now must be extending the evidence base for microbiome interventions from studies in healthy, non-psychiatric populations to people with clinically diagnosed anxiety disorders, and with an increased focus on function over form. This applies to the spectrum of microbiome-based interventions, including probiotics, prebiotics, synbiotics, whole-diet interventions and individual dietary components such as fermented foods. There is reasonably robust evidence for using specific adjunctive probiotics in patients with MDD [56, 83, 84]. Additionally, a Mediterranean diet intervention can also be recommended to depressed patients [85]. However, evidence is lacking in patients with clinical anxiety disorders and although similar interventions do hold promise, they cannot confidently be recommended by psychiatrists at present.

Adequately powered clinical trials in well-characterised groups of people with GAD, PD, agoraphobia and SAD are required to investigate the therapeutic potential of microbiome-based interventions. The many confounding factors which influence the human gut microbiome must be accounted for including diet, psychotropic and other medications, smoking, alcohol use and body mass index. An additional avenue for future research is clarifying the mechanisms underlying the mental health benefit of such interventions. Further exploration of the impact of probiotics, dietary components such as fermented foods and bacterial metabolites such as short-chain fatty acids on HPA axis function [86, 87], immune response, tryptophan metabolism, vagal nerve communication, BDNF expression, blood–brain-barrier integrity and other physiological processes involved in brain function and mental health is needed. Moreover, while much attention has focused on gut bacteria, the gut virome is also an important component of the human microbiome. It was recently demonstrated in rodents that the virome plays a role in the modulation of the microbiota–gut–brain axis during stress [88] indicating that viral populations should be considered when designing future microbiome-directed therapies.

The microbiome-gut-brain axis may be a promising new therapeutic target for the millions of people worldwide suffering from anxiety disorders. However, it has been a neglected topic of research in clinical anxiety disorder cohorts despite the promising preclinical signals, which were among the first to be noted in the field. It will be important to parse the common or distinct roles of the microbiome in clinically-diagnosed anxiety disorders as well as in those with high trait anxiety as a risk factor in otherwise healthy individuals and in people with comorbid anxiety in psychiatric and other disorders. The limitation of a symptomatic ‘floor effect’ when exploring the anxiolytic properties of microbiome interventions in healthy non-clinical populations may have underestimated the potential of this option and strain-specific effects also need to be taken into account. It is time for microbiome researchers to turn their attention towards people suffering with GAD, PD, agoraphobia and SAD, as well as those with sub-threshold anxiety symptoms, in order to fully elucidate the potential of the microbiome-gut-brain axis in such conditions.

## Key References

- **Nikolova, V.L., et al., *Perturbations in Gut Microbiota Composition in Psychiatric Disorders: A Review and Meta-analysis.* JAMA Psychiatry, 2021. 78(12): p. 1343–1354.**
  - A systematic review and meta-analysis of gut microbiota alterations in general adult psychiatric populations. Authors reported a transdiagnostic pattern with a depletion of certain anti-inflammatory butyrate-producing bacteria and an enrichment of pro-inflammatory bacteria in patients with depression, bipolar disorder, schizophrenia, and anxiety.
- **Brushett, S., et al., *Gut feelings: the relations between depression, anxiety, psychotropic drugs and the gut microbiome.* Gut Microbes, 2023. 15(2): p. 2281360.**
  - A large cohort study which analyzed data from 7,656 participants of the Dutch Lifelines population cohort to explore associations of the gut microbiome with depressive and anxiety disorders, with adjustment for use of psychotropic drugs.
- **Ritz, N.L., et al., *Social anxiety disorder-associated gut microbiota increases social fear.* Proceedings of the National Academy of Sciences, 2024. 121(1): p. e2308706120.**
  - A study which demonstrated that transplantation of the faecal microbiota from patients with social anxiety disorder to mice resulted in a distinct heightened social fear response in the recipient mice, coupled with changes in immmune function and central oxytocin expression.
- **Zhao, Z., et al., *Effectiveness of probiotic/prebiotic/synbiotic treatments on anxiety: A systematic review and meta-analysis of randomized controlled trials.* Journal of Affective Disorders, 2023. 343: p. 9–21.**
  - A systematic review and meta-analysis of the the effects of probiotics, prebiotics, and synbiotics on anxiety symptoms.
- **Wastyk, H.C., et al., *Gut-microbiota-targeted diets modulate human immune status.* Cell, 2021. 184(16): p. 4137–4153.e14.**
  - A 17-week randomized, prospective study which investigated the impact of two microbiota-targeted dietary interventions, plant-based fiber and fermented foods, on the human microbiome and immune system in healthy adults.
- **Ghosh, T.S., et al., *Mediterranean diet intervention alters the gut microbiome in older people reducing frailty and improving health status: the NU-AGE 1-year dietary intervention across five European countries.* Gut, 2020. 69(7): p. 1218.**
  - A large multicentre trial across five European countries which investigated the impact of a 1-year Mediterranean diet intervention on the gut microbiota and frailty status in elderly people.
- **Ritz, N.L., et al., *The gut virome is associated with stress-induced changes in behaviour and immune responses in mice.* Nature Microbiology, 2024. 9(2): p. 359–376.**
  - A mouse study demonstrating that the gut virome plays a role in the modulation of the microbiota–gut–brain axis during stress.

## Data Availability

No datasets were generated or analysed during the current study.
